# Dietary Saturated Fatty Acid Intake and Early Age-Related Macular Degeneration in a Japanese Population

**DOI:** 10.1167/iovs.61.3.23

**Published:** 2020-03-17

**Authors:** Mariko Sasaki, Sei Harada, Kazuo Tsubota, Tsutomu Yasukawa, Toru Takebayashi, Yuji Nishiwaki, Ryo Kawasaki

**Affiliations:** 1 Department of Ophthalmology, Keio University School of Medicine, Tokyo, Japan; 2 Tachikawa Hospital, Tokyo, Japan; 3 National Institute of Sensory Organs, National Tokyo Medical Center, Tokyo, Japan; 4 Department of Preventive Medicine and Public Health, Keio University School of Medicine, Tokyo, Japan; 5 Department of Ophthalmology and Visual Science, Nagoya City University Graduate School of Medical Sciences, Aichi, Japan; 6 Department of Environmental and Occupational Health, Toho University, Tokyo, Japan; 7 Department of Vision Informatics (Topcon), Osaka University Graduate School of Medicine, Osaka, Japan

**Keywords:** age-related macular degeneration, fatty acids, dietary intake

## Abstract

**Purpose:**

To assess the association of dietary saturated fatty acid (SFA) intake with the presence of early AMD in a Japanese population.

**Methods:**

The population-based Tsuruoka Metabolomics Cohort Study enrolled general population individuals aged 35 to 74 years from among participants in annual health check-up programs that included fundus photographs in Tsuruoka, Japan. A total of 4010 individuals participated in the baseline survey. After excluding nonresponders to a dietary survey and participants with suboptimal fundus image quality, 3988 participants (median age, 62.4 years) were included in this cross-sectional analysis. Dietary intake was assessed by a validated food frequency questionnaire. Fatty acids intake was adjusted for total energy intake by the residuals method. The association between fatty acid intake and presence of early AMD was assessed by logistic regression models.

**Results:**

Median daily SFA intake was 11.3 g (interquartile range, 9.6, 13.0 g). After adjustments for potential confounding factors, participants in the highest quartile of SFA intake were less likely to have early AMD, compared with the lowest quartile (odds ratio, 0.71; 95% confidence interval: 0.52–0.96). A significant trend for decreased risk of early AMD with increasing SFA intake was noted (*P* = 0.011). There was no significant association between poly-unsaturated fatty acid (PUFA) including n3-PUFA intake and early AMD.

**Conclusions:**

We found that increased SFA intake was associated with reduced risk of early AMD in a Japanese population with low SFA intake. Adequate fatty acid intake may be required to maintain retinal homeostasis and prevent AMD.

AMD is a leading cause of visual loss among elderly people worldwide,^1^ including in Japanese and other Asian populations.^2^ AMD is becoming an increasingly important health care problem in Asia, because Asians currently comprise 60% of the world's population and are projected to contribute the highest global prevalence of AMD by 2040.^3^

Soft drusen are well known to be lipid-rich and a main constituent of early AMD. The retinal pigment epithelium (RPE) persistently secretes apoB/apoE-containing lipoproteins, which accumulate on the surface of the Bruch membrane.^4^^,^^5^ Because the oxidation or modification of these lipoproteins is associated with the formation of soft drusen,^6^ dietary intake of fatty acids could influence lipid metabolism and the pathogenesis of early AMD.

A number of epidemiological studies have indicated that dietary intake of n-3 polyunsaturated fatty acids (PUFA) and fish could reduce AMD risk.^7^^–^^10^ A meta-analysis showed that intake of n-3 PUFA may reduce the risk of late AMD, and fish consumption may reduce the risk of both late and early AMD.^11^ However, data regarding the association between AMD and saturated fatty acid (SFA) intake are limited and inconsisitent.^12^^–^^17^

The associations between dietary fatty acid intake and AMD have been studied in Western populations; however, few such studies have been conducted among Asians,^2^ especially for early AMD. Besides, joint effects on AMD by fatty acid intake and patient genotype^18^^–^^21^ have been suggested. Because dietary patterns and genotypes differ between Western and Asian populations, the influence of fatty acid intake on AMD may also be different between these populations. We therefore aimed to examine the cross-sectional associations of dietary fatty acid intake with early AMD in a Japanese cohort from the Tsuruoka Metabolomics Cohort Study.

## Methods

### Study Population

This study was based on data derived from participants in the Tsuruoka Metabolomics Cohort Study, details of which have been previously described.^22^^,^^23^ In brief, from April 2012 through March 2015, 71,868 residents aged 35 to 74 years and enrolled in national health insurance in Tsuruoka, Japan, were identified and invited to participate in annual municipal health checkup programs that included fundus photographs. Of these, 8415 participants (11.7%) were enrolled. The present analysis included a total of 4010 individuals who participated in the baseline survey between April 2012 and March 2013.

This study was conducted in accordance with the Ethical Guidelines for Medical and Health Research Involving Human Subjects, Japan, and approved by the Medical Ethics Committees of the School of Medicine, Keio University, Tokyo, Japan (Approval No. 20110264), and the School of Medicine, Toho University, Tokyo, Japan (Approval No. 26028). Written informed consent was obtained from all participants included in the study.

### Data and Sample Collection

Each participant underwent a comprehensive assessment including a range of clinical, biochemical, and anthropometric measurements. Lifestyle factors were assessed using validated questionnaires. All data and samples were obtained during annual health checkups.

Nonstereoscopic fundus photographs of one eye (generally the right eye) were obtained using a 45° nonmydriatic fundus camera (TRC-NW200; TOPCON Corporation, Tokyo, Japan) without use of pharmacological dilating agents. Images were centered on the optic disc and macula. If fundus photography of the right eye was not possible because of media opacity or other reasons, the left eye was photographed (n = 126).

Blood pressure (BP) was measured twice after participants had been seated comfortably for at least 5 minutes. The means of two measures of systolic and diastolic BP were used for analysis. Body mass index (BMI) was calculated as weight (kg) divided by the height squared (m^2^). Blood samples were collected between 8:30 AM and 10:30 AM after overnight fasting to avoid variation due to fasting state and circadian rhythm. Blood glucose, hemoglobin A1c (%), and lipids (total cholesterol [TC], HDL-C, LDL-C, and triglyceride [TG]; mg/dL) were measured. Serum lipid levels were measured from fasting blood samples using an enzyme assay confirmed by the Japan Medical Association to be precise and valid for standardized testing.^24^ The following reagents were used for the Tsuruoka Study: TC Reagent: “L-type wako CHO M” (Wako Pure Chemical Industries, Ltd.), HDL-C Reagent: “MetaboRead HDL-C2” (Kyowa Medex Co., Ltd.), TG Reagent: “Determinar LTG II (Kyowa Medex Co., Ltd.). LDL-C levels were calculated using the Friedenwald formula when plasma TG concentrations were ≤400 mg/dL. Twenty-eight participants were excluded for having TG concentrations above this threshold.

Information regarding smoking history, alcohol intake, and use of antihypertension, antidiabetic, and lipid-lowering medications was obtained using a standardized self-administered questionnaire. Hypertension was defined as systolic BP ≥140 mm Hg, diastolic BP ≥90 mm Hg,^25^ or use of antihypertension medication. Diabetes was defined as hemoglobin A1c (National Glycohemoglobin Standardization Program) ≥6.5% or use of antidiabetic medication. Dyslipidemia was defined as LDL-C ≥140 mg/dL, HDL-C <40 mg/dL, TG ≥150 mg/dL, or use of lipid-lowering medication.

Dietary intake was assessed using a short food frequency questionnaire (FFQ).^26^ The dietary data collected included habitual dietary intake and frequency during the previous year for 47 foods/recipes; the portion size/serving size was requested for staple foods. Fatty acid intake in the FFQ was validated against that in the 3-day weight diet records as a reference,^27^^,^^28^ and satisfactorily high relative validity indices were attained. This suggests that the FFQ is valid and applicable, especially for categorizing individuals and determining fatty acid intake in dietary studies.^27^

### Grading of Fundus Photographs

A nonmydriatic fundus photograph of one eye of each participant was evaluated to determine whether quality was sufficient for grading of AMD lesions. Fundus photographs were evaluated at the reading center of Yamagata University (principal investigators: RK and YK). AMD photograph grading was performed according to protocols used in the Blue Mountains Eye Study, described in detail elsewhere.^29^ In brief, a trained grader (YK) assessed photographs for AMD signs in a masked fashion, following the modified Wisconsin Age-Related Maculopathy Grading System^30^ protocol used in the Blue Mountains Eye Study,^29^ with consultation from a senior researcher (RK) when grading uncertainties occurred.

Early AMD was defined as the presence of a large drusen (soft distinct or soft indistinct drusen) with a diameter >125 µm and/or RPE abnormality (hyperpigmentation or hypopigmentation) within the grid (a 3000-µm radius centered on the fovea), in the absence of late AMD.^30^^,^^31^ Late AMD was defined as the presence of exudative AMD or geographic atrophy. Exudative AMD was defined as the presence of subretinal or sub-RPE hemorrhage, RPE detachment, serous detachment of the sensory retina, or subretinal fibrous scars.^31^ Geographic atrophy was defined as sharply edged, roughly round, or oval areas of RPE loss, with clearly visible choroidal vessels.^31^

### Genotyping

Genomic DNA was extracted from the buffy coat fraction in accordance with standard procedures using a phenol–chloroform extraction method and checked for quality using Qubit (Life Technologies).

Participants were tested for two major AMD-associated SNPs: *ARMS2 A69S* (rs10490924) and *CFH I62V* (rs800292) using SNP Type Assays (Fluidigm, San Francisco, CA, USA). The quality control of genotyping was assessed statistically using the Hardy-Weinberg test, and *P* values > 0.05 were considered that genotype distributions were in Hardy-Weinberg Equation. Five percent random-samples were retyped by two different examiners, and those were 100% matched.

### Statistical Analysis

Baseline participant characteristics were summarized and stratified according to presence of early AMD. Differences between controls and subjects with early AMD were assessed using the Wilcoxon rank-sum test for continuous variables and the chi-squared test for categorical variables. Dietary intakes of total fat, SFA, monounsaturated fatty acids, PUFA, n-3 PUFA, n-6 PUFA, n-3 highly unsaturated fatty acids, and vitamins (vitamin C, vitamin E, and beta carotene) were adjusted for total energy intake using the residuals method as described by Willett and Stampfer^32^ and the sample median of 1738.5 kcals. Energy-adjusted fatty acid intake was then categorized into quartiles and analyzed with the lowest quartile as the reference group. Between-quartile differences in serum lipid levels were assessed using analysis of variance with the post hoc Dunnett test.

The association between fatty acid intake quartile and early AMD was assessed using three multivariable logistic regression models and expressed as odds ratios (ORs) with 95% confidence intervals (CIs). The first model was adjusted for age and sex. The second model was adjusted for age, sex, BMI, smoking history, hypertension, dyslipidemia, and diabetes. The third model was adjusted for the covariates in the second model plus vitamin C, vitamin E, and beta carotene. An interaction term, created by multiplying the SFA intake and patient genotypes (ARMS2 A69S and CFH I62V), was added to the model to assess statistical interactions and joint effect. Associations between the presence of early AMD and genetic factors were assessed using multivariable logistic regression models adjusted for age and sex. *P* values < 0.05 were considered statistically significant. SAS, version 9.4, for Windows (SAS Institute, Inc., Cary, NC) was used to perform all statistical analyses.

## Results

Of 4010 potential participants, 22 were excluded owing to missing fundus images or suboptimal fundus image quality (i.e., poor focus, eyelash artifacts, or uneven illumination); the remaining 3988 participants (99.5%) were included as subjects in this analysis. There were 1815 (45.5%) men and 2173 (54.5%) women; mean age was 62.4 ± 7.6 years. There were no differences in characteristics between those included in the analyses and those excluded (data not shown).

Baseline characteristics according to the presence of early AMD are presented in Table 1. Mean total daily SFA intake was 11.6 ± 2.7 g, and median (range) intake was 11.3 (9.6-13.0) g (6.0 ± 1.7 %energy and 5.9 [4.9-6.9] %energy, respectively). SFA intake was significantly different between controls and subjects with early AMD (*P* = 0.017). Subjects with early AMD were significantly older than controls (*P* < 0.0001) and were more likely to have hypertension (*P* = 0.014), use of antihypertension medication (*P* = 0.024), higher HDL-C (*P* = 0.043), and higher total calorie intake (*P* = 0.002).

**Table 1.** Baseline Characteristics Stratified by the Presence of Early AMD

	No. (%) or Mean (Standard Deviation)			
Variables	All Subjects	Control	Early AMD	*P* for Difference
**N**	3998	3541	447	
**Age, y**	62.4 (7.6)	62.0 (7.7)	65.3 (6.3)	**<** **0** **.0001**
**Sex, % male**	1815 (45.5)	1591 (44.9)	224 (50.1)	**0.038**
**BMI, kg/m^2^**	23.3 (3.2)	23.3 (3.3)	23.4 (3.0)	0.136
**Smoking status, %** **current**	590 (14.8)	526 (14.9)	64 (14.3)	0.763
**Hypertension**	1994 (50.0)	1746 (49.3)	248 (55.5)	**0.014**
**Dyslipidemia**	2093 (52.5)	1855 (52.4)	238 (53.2)	0.732
**Diabetes**	395 (9.9)	357 (10.1)	38 (8.5)	0.292
**Anti** **hypertension medication**	1250 (31.3)	1089 (30.8)	161 (36.0)	**0.024**
**Lipid-lowering medication**	828 (20.8)	723 (20.4)	105 (23.5)	0.131
**Total cholesterol, mg/dL**	210.4 (33.6)	210.5 (33.6)	210.1 (33.0)	0.817
**HDL, mg/dL**	68.4 (17.2)	68.3 (17.4)	69.6 (16.3)	**0.043**
**LDL cholesterol, mg/dL**	120.3 (30.3)	120.3 (30.2)	120.1 (31.0)	0.939
**Triglycerides, mg/dL**	110.2 (74.0)	111.2 (76.2)	102.4 (52.5)	0.120
**Total calorie, kcal**	1799.6 (415.5)	1794.1 (414.8)	1842.8 (418.4)	**0.002**
**Total fat, g**	45.2 (13.0)	45.2 (13.0)	44.6 (13.2)	0.057
**SFA, g**	11.6 (2.7)	11.6 (2.7)	11.3 (2.5)	**0.017**
**MUFA, g**	17.4 (4.8)	17.4 (4.8)	17.2 (4.8)	0.284
**PUFA, g**	15.5 (4.5)	15.5 (4.5)	15.6 (4.5)	0.771
**n3-PUFA, g**	2.6 (0.8)	2.6 (0.8)	2.6 (0.8)	0.825
**n6-PUFA, g**	12.8 (4.0)	12.8 (4.0)	12.8 (3.8)	0.927
**n3-HUFA, g**	1.0 (0.5)	0.9 (0.5)	1.0 (0.5)	0.076
**Alcohol intake, g**	12.8 (22.0)	12.8 (22.1)	13.4 (20.9)	0.360
**Beta-carotene, µg**	3631.9 (1752.3)	3621.4 (1715.0)	3714.5 (2023.8)	0.807
**Vitamin C intake, mg**	96.8 (42.6)	96.4 (40.9)	100.4 (54.5)	0.219
**Vitamin E intake, mg**	8.8 (2.7)	8.8 (2.6)	8.9 (2.9)	0.798

Significant values are in bold.

Fatty acids and micronutrients intake are expressed as energy adjusted values.

HUFA, highly unsaturated fatty acid; MUFA, monounsaturated fatty acid; PUFA, polyunsaturated fatty acid; SFA, saturated fatty acid.

After adjusting for age, sex, BMI, smoking history, hypertension, dyslipidemia, diabetes, beta carotene, vitamin C, and vitamin E, subjects in the highest quartile of SFA intake were less likely to have early AMD compared with those in the lowest quartile (OR, 0.71, 95% CI: 0.52-0.96). A significant trend of decreasing OR for AMD with increasing SFA intake was noted (*P* = 0.011) (Table 2). The results of the association between fatty acid intake and the prevalence of early AMD were not markedly changed when using %kcals instead of the residual method (data not shown). There was no association between other fatty acid types, including n-3 PUFA, and presence of early AMD. Furthermore, when participants with co-morbidities that might have influenced previous dietary changes were removed from the analyses, the results were not substantially changed (*P* for trend: 0.06 without dyslipidemia and 0.03 without diabetes).

**Table 2.** The Association Between Fatty Acid Intake and the Presence of Early AMD

Fatty Acid Intake	Quartile 1	Quartile 2	Quartile 3	Quartile 4	*P* for Trend
N	997	997	997	997	
Age (mean ± SD)	61.7 ± 7.7	61.9 ± 8.0	63.1 ± 7.2	62.8 ± 7.4	
**Total fat**					
Median (range) intake, energy adjusted, g	32.5 (16.2, 36.8)	40.2 (36.8, 43.5)	46.8 (43.5, 51.2)	57.3 (51.2, 162.7)	
No. with outcome/at risk	124/997	117/997	106/997	100/997	
Model 1, OR (95% CI)	Reference	0.94 (0.72–1.24)	0.87 (0.65–1.16)	0.83 (0.62–1.12)	0.185
Model 2, OR (95% CI)	Reference	0.94 (0.72–1.24)	0.87 (0.66–1.16)	0.83 (0.62–1.12)	0.186
Model 3, OR (95% CI)	Reference	0.90 (0.68–1.20)	0.81 (0.59–1.09)	0.70 (0.49–1.02)	0.053
**Saturated fatty acids**					
Median (range) intake, energy adjusted, g	8.7 (5.7–9.6)	10.4 (9.6–11.3)	12.1 (11.3–13.0)	15.1 (13.0–27.6)	
No. with outcome/at risk	126/997	119/997	106/997	96/997	
Model 1, OR (95% CI)	Reference	0.94 (0.72–1.24)	0.78 (0.59–1.03)	**0.73 (0.54** **–** **0.98)**	**0.016**
Model 2, OR (95% CI)	Reference	0.95 (0.72–1.25)	0.78 (0.59–1.04)	**0.73 (0.55** **–** **0.98)**	**0.017**
Model 3, OR (95% CI)	Reference	0.94 (0.71–1.24)	0.77 (0.58–1.03)	**0.71 (0.52** **-** **0.96)**	**0.011**
**Monounsaturated fatty acids**					
Median (range) intake, energy adjusted, g	12.8 (8.1–14.1)	15.3 (14.1–16.7)	18.1 (16.7–19.7)	22.3 (19.7–75.7)	
No. with outcome/at risk	114/997	108/997	129/997	96/997	
Model 1, OR (95% CI)	Reference	0.98 (0.74–1.31)	1.29 (0.97–1.70)	0.93 (0.69–1.25)	0.885
Model 2, OR (95% CI)	Reference	0.99 (0.74–1.31)	1.29 (0.98–1.71)	0.93 (0.69–1.25)	0.876
Model 3, OR (95% CI)	Reference	0.97 (0.72–1.29)	1.24 (0.91–1.68)	0.85 (0.57–1.26)	0.955
**Polyunsaturated fatty acids**					
Median (range) intake, energy adjusted, g	10.9 (5.1–12.4)	13.7 (12.4–15.1)	16.3 (15.1–17.8)	20.3 (17.8–66.7)	
No. with outcome/at risk	112/997	113/997	104/997	118/997	
Model 1, OR (95% CI)	Reference	0.98 (0.74–1.29)	0.93 (0.69–1.24)	1.03 (0.77–1.37)	0.906
Model 2, OR (95% CI)	Reference	0.97 (0.73–1.29)	0.93 (0.69–1.24)	1.03 (0.78–1.38)	0.889
Model 3, OR (95% CI)	Reference	0.97 (0.73–1.30)	0.92 (0.67–1.26)	1.02 (0.71–1.48)	0.968
**n3- polyunsaturated fatty acids**					
Median (range) intake, energy adjusted, g	1.8 (1.1–2.1)	2.3 (2.1–2.5)	2.7 (2.5–2.9)	3.3 (2.9–10.2)	
No. with outcome/at risk	112/997	107/997	110/997	118/997	
Model 1, OR (95% CI)	Reference	0.90 (0.68–1.21)	0.94 (0.70–1.25)	0.97 (0.73–1.30)	0.890
Model 2, OR (95% CI)	Reference	0.90 (0.67–1.20)	0.95 (0.71–1.26)	0.98 (0.73–1.30)	0.984
Model 3, OR (95% CI)	Reference	0.89 (0.66–1.19)	0.93 (0.69–1.27)	0.94 (0.66–1.35)	0.808
**n6- polyunsaturated fatty acids**					
Median (range) intake, energy adjusted, g	8.8 (3.9–10.1)	11.3 (10.1–12.4)	13.5 (12.4–14.9)	17.0 (14.9–64.8)	
No. with outcome/at risk	113/997	113/997	108/997	113/997	
Model 1, OR (95% CI)	Reference	1.00 (0.76–1.33)	0.98 (0.74–1.31)	1.02 (0.77–1.36)	0.921
Model 2, OR (95% CI)	Reference	1.00 (0.76–1.33)	0.98 (0.73–1.31)	1.02 (0.77–1.37)	0.924
Model 3, OR (95% CI)	Reference	1.00 (0.75–1.33)	0.97 (0.71–1.32)	1.00 (0.69–1.44)	0.938
**n3-highly unsaturated fatty acids**					
Median (range) intake, energy adjusted, g	0.5 (0.2–0.7)	0.8 (0.7–0.8)	1.0 (0.8–11.8)	1.3 (1.2–4.7)	
No. with outcome/at risk	112/997	100/997	100/997	135/997	
Model 1, OR (95% CI)	Reference	0.84 (0.63– 1.12)	0.78 (0.58–1.04)	0.95 (0.72–1.26)	0.733
Model 2, OR (95% CI)	Reference	0.83 (0.62–1.12)	0.78 (0.58–1.04)	0.97 (0.73–1.29)	0.826
Model 3, OR (95% CI)	Reference	0.83 (0.62–1.11)	0.77 (0.57–1.04)	0.95 (0.71–1.28)	0.720

Significant values are in bold.

Model 1: adjusted for age and sex.

Model 2: adjusted for age, sex, BMI, smoking history, hypertension, dyslipidemia, and diabetes.

Model 3: adjusted for age, sex, BMI, smoking history, hypertension, dyslipidemia, diabetes, beta-carotene, vitamin C, and vitamin E.

BMI, body mass index.

Table 3 shows genetic characteristics by SFA intake quartiles. In quartile (Q) 1, the ARMS2 A69S TT genotype was found in 13.5% of subjects and was associated with increased odds of early AMD (OR, 2.35; 95% CI: 1.29-4.29), whereas the CFH I62V CT and TT genotypes were found in 50.8% and 16.7% of subjects, respectively, and were also associated with increased risk (OR, 1.82 and 2.08, respectively; 95% CI: 1.07-3.10 and 1.08-4.03, respectively). Similar association was observed between the ARMS2 A69S or CFH I62V variants and early AMD in Q4. No interactions were found between SFA intake and these variants (*P* = 0.302 for ARMS2 A69S and *P* = 0.621 for CFH I62V).

**Table 3.** Genetic Risk for Early AMD Stratified by Saturated Fatty Acid Intake

	Quartile 1				Quartile 4			
		Control	Early AMD	Odds		Control	Early AMD	Odds
Genotypes	Prevalence (%)	No. (%)	No. (%)	(95% CI)	Prevalence (%)	No. (%)	No. (%)	(95% CI)
**rs10490924/ ARMS2 A69S**								
GG	39.9	282 (89.2)	34 (10.8)	1 (reference)	37.1	274 (92.6)	22 (7.4)	1 (reference)
TG	46.6	325 (88.1)	44 (11.9)	1.14 (0.70–1.83)	48.6	351 (90.5)	37 (9.5)	1.31 (0.75–2.30)
TT	13.5	85 (79.4)	22 (20.6)	**2.35 (1.29**–**4.29)**	14.4	99 (86.1)	16 (13.9)	1.92 (0.96–3.86)
**rs800292/ CFH I62V**								
CC	32.6	237 (91.9)	21 (8.1)	1 (reference)	36.4	268 (92.1)	23 (7.9)	1 (reference)
CT	50.8	343 (85.3)	59 (14.7)	**1.82 (1.07** **–** **3.10)**	46.9	343 (91.5)	32 (8.5)	1.01 (0.57–1.78)
TT	16.7	112 (84.9)	20 (15.2)	**2.08 (1.08** **–** **4.03)**	16.7	113 (85.0)	20 (15.0)	1.88 (0.98–3.61)

Adjusted for age and sex; significant values are in bold.

Finally, the association of SFA intake with serum lipid levels is presented in Table 4. Serum TC and LDL-C levels were significantly higher in the other quartiles of SFA intake compared with Q1. Similarly, serum HDL-C levels were significantly higher in Q3 and Q4 than Q1. On the other hand, serum triglyceride levels were significantly lower in Q3 and Q4 compared with Q1. The TC to HDL-C ratio was significantly lower in Q4 than Q1.

**Table 4.** Serum Lipid Levels Stratified by Saturated Fatty Acid Intake

	SFA Intake				
Serum Lipid Levels	Quartile 1 (Reference)	Quartile 2	Quartile 3	Quartile 4	*P* for Difference Across Quartiles
**Total cholesterol, mg/dL**	206.7 (34.9)	**211.2 (32.4)**	**210.5 (32.9)**	**213.4 (33.7)**	0.0001
**HDL-cholesterol, mg/dL**	66.5 (17.8)	67.8 (16.9)	**68.8 (16.7**)	**70.6 (17.2)**	<0.0001
**LDL-cholesterol, mg/dL**	116.9 (31.4)	**121.0 (29.6)**	**120.8 (29.5)**	**122.5 (30.3)**	0.0004
**Triglycerides, mg/dL**	118.6 (89.9)	115.1 (81.8)	**104.8 (58.5)**	**102.2 (59.3)**	<0.0001
**TC to HDLC ratio**	3.29 (0.91)	3.30 (0.94)	3.21 (0.84)	**3.18 (0.85)**	0.005

Adjusted for age and sex.

Differences were assessed using analysis of variance with the post hoc Dunnett test. Significant values against the first quartile are in bold.

TC, total-cholesterol.

## Discussion

In the current study, there was an inverse association between SFA intake and the presence of early AMD in a Japanese population. No significant association was found between intake of PUFA, including n-3 PUFA, and the presence of early AMD. Interestingly, SFA intake influenced serum lipid levels and was associated with improved ratio of TC to HDL-C, which is a strong risk marker of coronary heart disease (CHD). There were no interactions between SFA intake and genotypes.

Epidemiological studies and meta-analysis have suggested that dietary intake of n-3 PUFA and fish could reduce the risks of early and late AMD.^7^^–^^11^ However, in the present study, there was no significant association between n-3 PUFA intake and the presence of early AMD. Previous reports indicate that beneficial effects on AMD risk were seen with at least one serving of fish per week.^8^^,^^14^^,^^33^ In Japan, approximately 90% of individuals consume fish one to two times per week.^34^^,^^35^ In fact, in the Melbourne Collaborative Cohort Study,^36^ median n3-PUFA intake was 1.0 and 1.4 g/d (energy-adjusted values) in the lowest and highest quartiles, respectively. In the current study, median n3-PUFA intake was 1.8 and 3.3 g/d in the lowest and highest quartiles, respectively. The lack of association we observed between n-3 PUFA intake and the presence of early AMD may have been due in part to this high amount of n3-PUFA intake. However, in a case-control study, dietary n-3 PUFA intake was reported to be associated with reduced risk of neovascular AMD in Japan. Further studies are needed in the Asian population.^37^

Studies of the associations between SFA intake and AMD risks are limited, and results have been inconsisitent.^12^^–^^17^ Studies have suggested that high SFA intake is associated with increased risk of AMD,^12^^,^^13^^,^^16^ whereas a few studies have suggested the opposite.^15^^,^^17^ Compared with Western populations, Asian populations consume less SFA-containing foods^38^^,^^39^; indeed, SFA intake in the Japanese population is thought to be approximately half that of Americans.^40^^,^^41^ In the Blue Mountains Eye Study, SFA intake averaged 20.1 g/d and 39.9 g/d (energy-adjusted values) in the lowest and highest quartiles, respectively.^15^ The Japan Collaborative Cohort Study for Evaluation of Cancer Risk Study reported that median SFA intake was 9.2 and 20.3 g/d (energy-adjusted values) in the lowest and highest quintiles, respectively.^42^ Likewise, in the current study, median SFA intake was 8.7 and 15.1 g/d in the lowest and highest quartiles, respectively. The very low SFA intake among our study subjects compared with Western study populations may account for the disparate results. Our findings suggest that the association may not be linear and may perhaps be a U-shaped curve instead. Further studies are needed to examine SFA in pooled studies with broader ranges in intake to understand the association. Because lipid metabolism can efficiently create energy through fatty acid β-oxidation in the retina,^43^^,^^44^ the maintenance of retinal homeostasis and prevention of AMD may require adequate fatty acid intake. Although joint effects on AMD by fatty acid intake and patient genotype has been suggested,^18^^–^^21^ there were no interactions between SFA intake and genotype in the current study.

It is well known that SFA intake increases total cholesterol levels,^45^ a risk factor for ischemic heart disease.^46^ A meta-analysis found that CHD was reduced by 10% for each 5% of energy from SFA that was replaced by energy from PUFA.^47^ SFA intake therefore has generally been considered to be atherogenic, although recent meta-analyses have not supported adverse effects of SFA intake on ischemic heart disease risk.^48^^,^^49^ On the other hand, SFA intake is known to raise HDL-C levels—an antiatherogenic effect^50^—and was reported to be inversely associated with the progression of coronary atherosclerosis.^51^ In the current study, subjects in the highest SFA intake quartile had higher levels of TC, HDL-C, and LDL-C compared with the lowest quartile. Notably, the TC to HDL-C ratio, which is a more global marker of CHD risk than TC or LDL-C, was improved in the highest quartile compared with the lowest. These findings suggest that increased SFA intake may not be atherogenic, at least in this Japanese cohort.

The strengths of this study include its large sample size, use of standardized grading protocols to define AMD by trained graders, and use of validated questionnaires to gather lifestyle and medical history information. We also recognize several limitations with our study. First, the study had a cross-sectional observational design, without temporal information regarding associations. Second, because there were few participants with late AMD due to the age range of the study population (35–74 years old) or potential healthy screenee bias, we were unable to analyze associations with late AMD. Third, the participation rate was relatively low, especially among residents younger than 60 years of age. The age distribution of the participants in this study was somewhat different from that of residents in Tsuruoka City overall. These factors might have affected the results of the diet assessment and prevalence of early AMD. Forth, food frequency was reported instead of specific quantities due to the difficulty of quantitative measurement, the intakes of micro- and macronutrients were already validated though. In the future, investigating the distribution of AMD grades according to serum fatty acid levels would provide important insight into our findings. Finally, lutein, zeaxanthin, and chain-length-specific effects of fatty acids on AMD could not be analyzed. Therefore, further study is needed to validate these findings.

In a large Japanese cohort, increased intake of SFA was associated with reduced risk of early AMD among subjects with very low SFA intake, whereas no significant association was found between PUFA intake and early AMD. Moreover, SFA intake influenced serum lipid levels and was associated with improved ratio of TC to HDL-C. Our findings suggest that the effects of SFA intake on AMD likely differ among populations with different lifestyles, and the association may not be linear. Adequate fatty acid intake may be necessary to maintain retinal homeostasis and prevent AMD.
